# ACE2-Independent Bat Sarbecovirus Entry and Replication in Human and Bat Cells

**DOI:** 10.1128/mbio.02566-22

**Published:** 2022-11-21

**Authors:** Hua Guo, Ang Li, Tian-Yi Dong, Jia Su, Yu-Lin Yao, Yan Zhu, Zheng-Li Shi, Michael Letko

**Affiliations:** a CAS Key Laboratory of Special Pathogens and Biosafety, Wuhan Institute of Virology, Chinese Academy of Sciences, Wuhan, China; b University of Chinese Academy of Sciences, Beijing, China; c Paul G. Allen School for Global Health, Washington State University, Pullman, Washington, USA; Washington University School of Medicine

**Keywords:** sarbecovirus, SARS-related coronavirus, ACE2-independent, trypsin-dependent

## Abstract

Hundreds of sarbecoviruses have been found in bats, but only a fraction of them have the ability to infect cells using angiotensin-converting enzyme 2 (ACE2), the receptor for SARS-CoV and -2. To date, only ACE2-dependent sarbecoviruses have been isolated from field samples or grown in the laboratory. ACE2-independent sarbecoviruses, comprising the majority of the subgenus, have not been propagated in any type of cell culture, as the factors and conditions needed for their replication are completely unknown. Given the significant zoonotic threat posed by sarbecoviruses, cell culture models and *in vitro* tools are urgently needed to study the rest of this subgenus. We previously showed that the exogenous protease trypsin could facilitate cell entry of viral-like particles pseudotyped with spike protein from some of the ACE2-independent sarbecoviruses. Here, we tested if these conditions were sufficient to support bona fide viral replication using recombinant bat sarbecoviruses. In the presence of trypsin, some of the spike proteins from clade 2 viruses were capable of supporting bat sarbecovirus infection and replication in human and bat cells. Protease experiments showed a specific viral dependence on high levels of trypsin, as TMPRSS2 and furin had no effect on clade 2 virus entry. These results shed light on how sarbecoviruses transmit and coexist in their natural hosts, provide key insights for future efforts to isolate and grow these viruses from field samples, and further underscore the need for broadly protective, universal coronavirus vaccines.

## INTRODUCTION

Sarbecoviruses are a subgenus of the betacoronaviruses and include the human pathogens severe acute respiratory syndrome coronavirus (SARS-CoV) and SARS-CoV-2, as well as hundreds of poorly characterized viruses found predominantly in bats around the world (1 to 17). Although next-generation sequencing technologies continue to improve, yielding even more viral sequences from wildlife samples, laboratory tools to study these viruses are severely lacking. Most viruses discovered to date have not been studied in a laboratory because the basic cell culture conditions necessary to grow the viruses *in vitro* are simply unknown. Field samples teeming with never-before-seen viral sequences often fail to produce physical virus, hindering our ability to study these viruses and greatly limiting our understanding of the natural virome and which members pose the greatest threat to global health.

**FIG 1:**
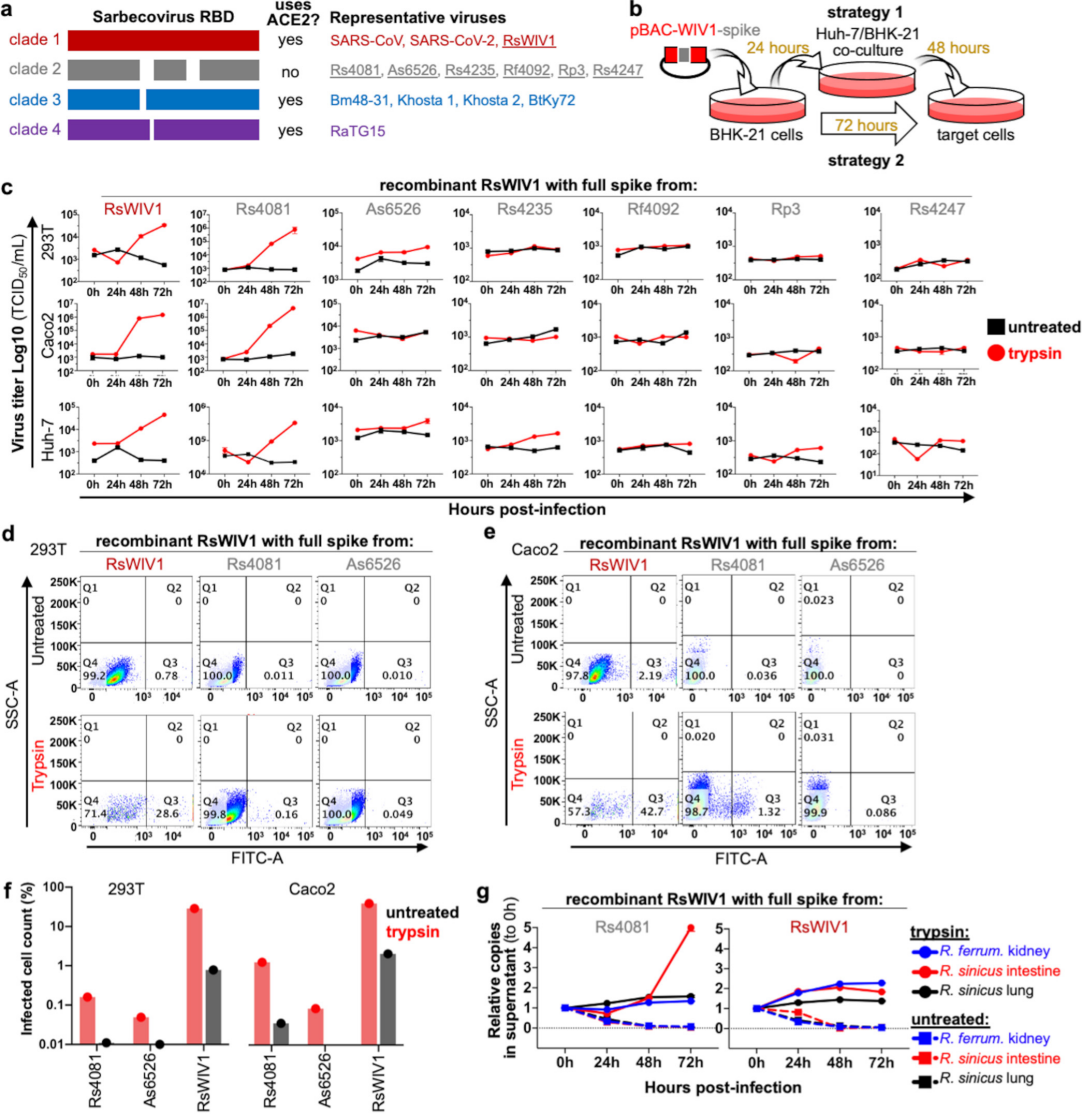
Exogenous trypsin mediates sarbecovirus RBD clade 2 replication in human and bat cells. (a) Receptor usage of the different clades of sarbecovirus RBD. (b) Outline of reverse genetics rescue strategies. (c) Viral replication on three different human cell lines was measured by RT-PCR for the RsWIV1 viral backbone (d) 293T or (e) Caco2 cells, which were infected, stained for viral nucleocapsid protein, and measured by fluorescence-activated cell sorter (FACS). (f) Quantification of FACS results. (g) Viral replication, with or without trypsin, on primary bat cell cultures. Error bars represent the corresponding mean ± SEM.

The spike (S) protein of coronaviruses mediates viral entry into host cells: the first and essential step for successful infection and transmission, both within and between species. Thus, the S protein is widely targeted for vaccine development and therapeutic interventions against SARS-CoV-2. The S gene is also one of the most variable regions in sarbecoviruses. Based on the receptor-binding domain (RBD) portion of spike and its subsequent receptor utilization, we and others have previously grouped sarbecovirus RBDs into at least 4 clades (10, 15, 18 to 21). Clade 1 sarbecoviruses like SARS-CoV, SARS-CoV-2, and the bat SARS-related coronavirus (SARSr-CoV) RsWIV1, contain no deletions and utilize the cell receptor angiotensin-converting enzyme 2 (ACE2) to enter host cells. Clade 2 sarbecoviruses contain two deletions in their RBD and do not use ACE2 for cell entry. Clade 3 and 4 sarbecoviruses contain one deletion and exhibit a partial capacity to utilize ACE2 from some species (Fig. 1a). Viruses from clade 1 have been widely studied using *in vitro* models with replicating viruses and pseudotypes in cell culture or *in vivo* animal models (8, 10, 18, 22 to 26). While clade 2 viruses were the first sarbecoviruses discovered in bats, initial attempts to isolate these viruses from field samples or propagate clade 2 viruses from reverse genetics systems were unsuccessful (1, 6, 10, 19, 21). To date, viruses from clades 2, 3, and 4 have only been assessed by protein-protein binding assays (measuring affinity of purified spike protein fragments for purified receptor binding fragments) or pseudotyped systems due to lack of live virus growth (15, 19 to 21).

Previously, we showed that viral-like particles pseudotyped with S from some of the clade 2 viruses could infect human cells in the presence of trypsin, demonstrating that clade 2 viruses may also pose a risk of cross-species transmission (19, 20). Although viral pseudotypes are generally a suitable model of true viral cell entry, there have been discrepancies between pseudotype and viral replication results for other bat sarbecoviruses. Moreover, the amount of trypsin we previously reported for clade 2 viral entry was higher than has been reported for other coronaviruses, leading us to wonder if a real virus could actually replicate under these conditions (19, 20). Here, we recovered replication-competent bat coronaviruses through reverse genetics approaches and confirm our earlier findings, demonstrating that these viruses are capable of replicating in a high-trypsin environment. Moreover, we explored the mechanistic aspects of trypsin’s role in viral entry and show that trypsin is not compensated by other known proteases involved in some coronavirus entry. As many of the enteric coronaviruses are known to require trypsin for entry, these findings provide valuable insights into what transmission pathways this curious group of potentially human-compatible sarbecoviruses may employ to persist in the host species.

## RESULTS

### Bat sarbecoviruses replicate on human and bat cells in the presence of trypsin.

Exogenous trypsin has been shown to facilitate viral cell entry for several different coronaviruses (19, 27 to 29), and we have previously shown that trypsin facilitates cell entry of vesicular stomatitis virus (VSV) pseudotyped particles bearing spike proteins from clade 2 RBDs (19, 30). To assess if trypsin-mediated entry was sufficient to support viral replication beyond just one cycle of cell entry, we generated recombinant sarbecovirus, replacing the spike gene in the bat SARSr-CoV virus, RsWIV1, with the spike gene from clade 2 viruses (Fig. 1a and b), as described previously (10). Using two different strategies for recombinant virus recovery (Fig. 1b), we found that some bat SARSr-CoVs belonging to clade 2 could be rescued in HEK293T, Caco2. or Huh-7 cell lines in the presence of trypsin, with spike from the clade 2 virus, Rs4081, mediating the clearest replication, similar to wild-type clade 1 virus RsWIV1 (Fig. 1c). We subsequently confirmed viral replication using a nucleocapsid (N) protein-specific antibody by flow cytometry and observed clear expression of N protein in cells from the trypsin-treated group following recombinant virus infection (Fig. 1d, e). Notably, the infection efficiency of the clade 1 virus, RsWIV1, was higher than clade 2 viruses in both HEK 293T and Caco2 cells (Fig. 1f). These recombinant viruses were subsequently verified by next-generation sequencing (NGS), and we did not observe any mutations in their genome (data not shown).

We next tested the capacity of the top-performing clade 2 chimeric virus to grow in bat cells. Recombinant virus rWIV1-Rs4081-S infected and replicated in Rhinolophus bat intestinal primary cells in the presence of trypsin, but not the primary cells from lung or kidney (Fig. 1g). Similar to what we and others have observed, the clade 1 RBD virus, RsWIV1, could not efficiently replicate in the primary Rhinolophus bat cell lines with or without exogenous trypsin treatment, although they can use the bat ACE2 for entry (Fig. 1g).

Taken together, these data demonstrate that exogenous trypsin imposes a broad effect, enhancing entry of both ACE2-dependent and ACE2-independent sarbecoviruses. In line with our previous findings, trypsin-mediated entry was specific for only some clade 2 viruses, but not others, suggesting viral replication was still dependent on the presence of a yet-uncharacterized receptor.

### ACE2-independent sarbecovirus infection depends on high levels of exogenous trypsin.

**FIG 2:**
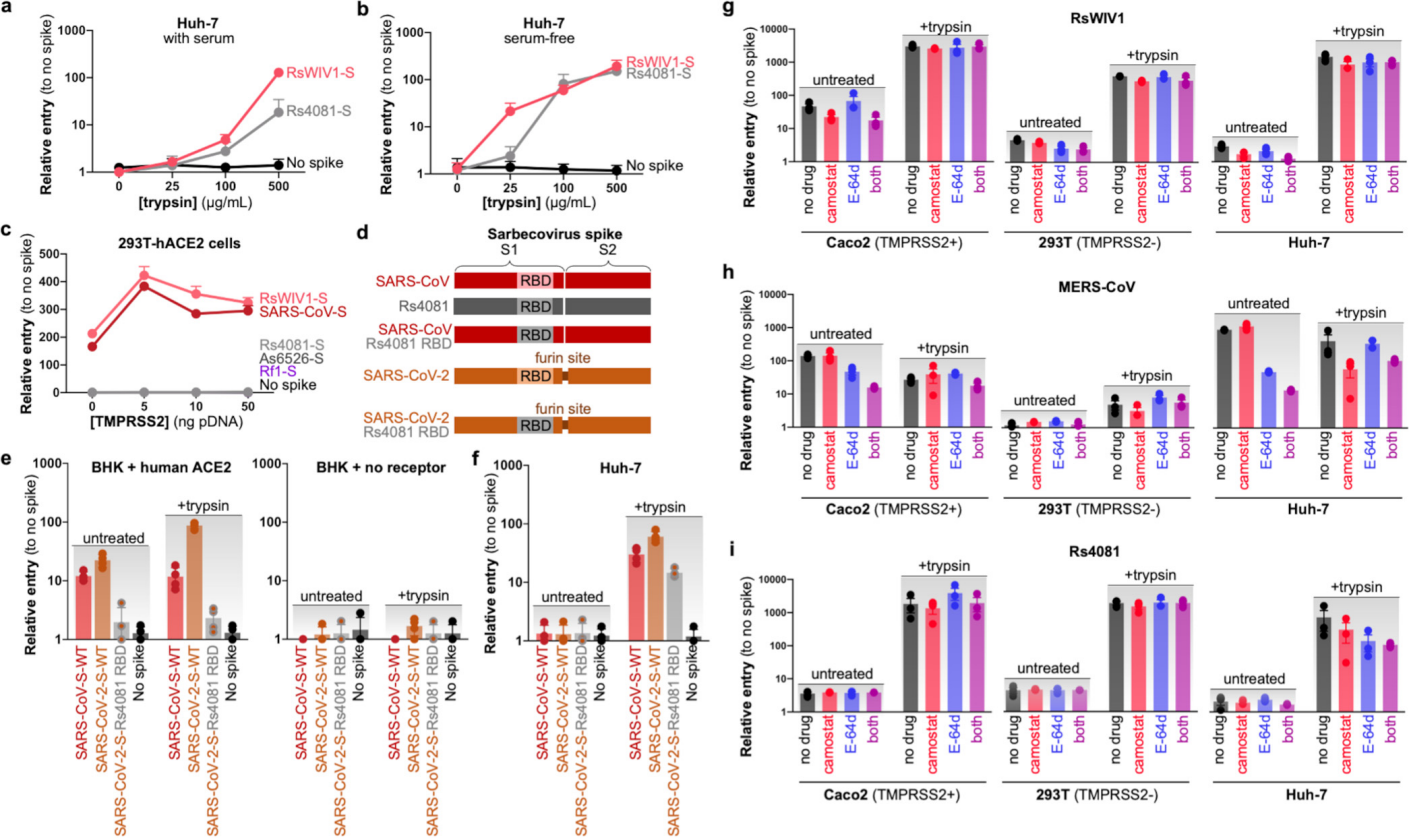
Sarbecovirus RBD clade 2 infection depends on high levels of trypsin. (a) Pseudotyped particles were collected in standard viral growth medium supplemented with 2% fetal bovine serum and used to infect Huh-7 cells with increasing amounts of trypsin. (b) Pseudotyped particles were collected in serum-free formulated viral growth medium and used to infect Huh-7 cells with increasing amounts of trypsin. (c) 293T cells stably expressing human ACE2 were transfected with increasing amounts of TMPRSS2 and infected with serum-free pseudotyped particles. (d) Pseudotypes were generated with a chimeric SARS-CoV-2 spike encoding the Rs4081 RBD and used to infect. (e) BHK cells transfected with human ACE2 or empty vector, or (f) Huh-7 cells, with or without trypsin. Shown are the data from quadruplicate infections. (g to i) Caco2, 293T, or Huh-7 were pretreated with the indicated protease inhibitors and infected with pseudotyped particles, with or without trypsin. Error bars represent the corresponding mean ± SEM.

In our previous studies and earlier experiments, the viral pseudotyped particles and replication-competent virus stocks were collected in media containing low levels of fetal bovine serum (FBS), which is well known to inhibit trypsin activity. To test if the trace levels of serum in our pseudotyped stocks accounted for the high concentration of trypsin required in our experiments thus far, we generated stocks with or without serum and performed entry assays with various levels of trypsin (Fig. 2a and b). Although eliminating serum did allow for lower concentrations of trypsin to recover pseudotype entry, there was still a noticeable difference between the clade 2 spikes and the clade 1 spikes at lower concentrations (Fig. 2b). Surprisingly, while 25 μg/mL was sufficient to activate the serum-free RsWIV1 or SARS-CoV spikes, this amount of trypsin was insufficient to recover clade 2 spikes, Rs4081 or As6526, which only entered cells at higher concentrations of trypsin (Fig. 2a and b; Fig. S1a and b in the supplemental material). Thus, while serum was obviously inhibiting trypsin in the viral stocks, the clade 2 spikes also seem to be inherently resistant to lower levels of trypsin sufficient to activate the clade 1 spikes. Similar to our previous studies, the GFP signal from the dual-reporter VSV pseudotype used in our assays was strong and increased over the course of infection, implying cell viability despite the prolonged trypsin exposure (19, 20).

10.1128/mbio.02566-22.1 FIG S1 Additional protease processing results related to Fig. 2. (a) Pseudotyped particles were collected in standard viral growth medium supplemented with 2% fetal bovine serum and used to infect Huh-7 cells with increasing amounts of trypsin. (b) Pseudotyped particles were collected in serum-free formulated viral growth medium and used to infect Huh-7 cells with increasing amounts of trypsin. (c to e) Caco2, 293T, or Huh-7 were pretreated with the indicated protease inhibitors and infected with pseudotypes, with or without trypsin. (f) Identical amounts of purified Rs4081 pseudotyped particles were incubated with different concentrations of trypsin at 37°C for 15 min. The cleavage of the spike was analyzed by Western blot using an anti-FLAG antibody, which targeted the C-terminal of spike, and the visualization of VSV backbone was detected by an anti-VSV matrix antibody. (g) Potential trypsin cleavage sites analysis using the PeptideCutter tool on the ExPASy Server (https://web.expasy.org/peptide_cutter/) and ProP1.0 software (https://services.healthtech.dtu.dk/service.php?ProP-1.0). Scores of >0.15 are listed. (h) Schematic of putative trypsin sites in Rs4081 spike with resulting C-terminal fragment and expected size indicated in kDa. Download FIG S1, JPG file, 0.9 MB. Copyright © 2022 Guo et al. 2022 Guo et al. https://creativecommons.org/licenses/by/4.0/ This content is distributed under the terms of the Creative Commons Attribution 4.0 International license.

Following receptor binding, sarbecoviruses such as SARS-CoV-2 subsequently enter host cells following either cell-surface or endosomal-pathway priming by transmembrane protease serine 2 (TMPRSS2) or cathepsin L, respectively (31, 32). To test if trypsin addition was compensating for a lack of endogenous TMPRSS2 expression, we overexpressed different concentrations of human TMPRSS2 in a HEK 293T cell line stably expressing human ACE2 and then transduced the cells with pseudotyped particles. In agreement with previous findings (31, 33), overexpression of human ACE2 and TMPRSS2 robustly enhanced the viral entry of clade 1 viruses. In contrast, coexpression of these two genes failed to mediate entry of any of the clade 2 viruses (Fig. 2c).

In addition to transmembrane-bound proteases directly involved in cell entry, furin has also been suggested to play a role in coronavirus entry. Most poignantly, SARS-CoV-2 spike contains a furin cleavage site that has been suggested to improve viral cell-surface entry kinetics (34). To test if a furin cleavage site could compensate for trypsin during cell entry, we generated virus-like particles pseudotyped with a SARS-CoV-2 chimeric spike containing the RBD from Rs4081 spike (Fig. 2d). Although wild-type SARS-CoV-2 spike could infect BHK cells overexpressing human ACE2, the chimeric SARS-CoV-2-Rs4081 RBD spike could not infect with or without trypsin (Fig. 2e). The chimeric clade 2 RBD spike was still capable of infecting the human Huh-7 cell line but only when trypsin was included, suggesting that the furin site was not modifying entry for the clade 2 virus RBD (Fig. 2f).

We next treated cells with camostat mesylate and E-64d, which inhibit TMPRSS2 or cathepsin B/L, respectively. HEK 293T cells are known to be low or negative for TMPRSS2 expression, while Caco2 cells express TMPRSS2 and can support viral entry at the cell surface (31). In agreement with previous findings on SARS-CoV and -2 (31, 33), we observed that in the absence of trypsin, entry for clade 1 virus and MERS-CoV was reduced by both inhibitors in combination (Fig. 2g to h; Fig. S1c to e). The clade 1 viruses were also sensitive to camostat mesylate alone, while MERS-CoV spike-driven entry was reduced by E-64d. The entry of negative-control VSV was not influenced by either of the inhibitors (Fig. 2g to h; Fig. S1e). In contrast, we did not observe significant effect with either inhibitor for any virus in the presence of the trypsin, except for the clade 2 virus Rs4081 and MERS-CoV, which showed a slight decrease by blocking with E-64d and camostat mesylate in combination in the Huh-7 cells, but not HEK 293T and Caco2 (Fig. 2h and i).

To further explore the effect of trypsin on Rs4081 spike, we incubated concentrated viral pseudotypes with increasing amounts of trypsin at 37^ο^C for 15 min and analyzed spike degradation by FLAG (Fig. S1f). Trypsin cleaved spike into several expected fragments corresponding to cleavage at the S1/S2 boundary as well as a secondary, S2’ site (Fig. S1f to h). At high concentrations, Rs4081 spike was quickly degraded, which is in line with other reports on coronavirus spikes incubated with trypsin for prolonged periods of time (29).

Taken together, the clade 2 viruses do not employ a combination of ACE2 and TMPRSS2 for human cell entry, but likely still use an entry pathway that partially overlaps with other known coronavirus mechanisms for membrane fusion.

### Purified clade 2 RBD protein directly binds to human cells in the absence of trypsin.

**FIG 3:**
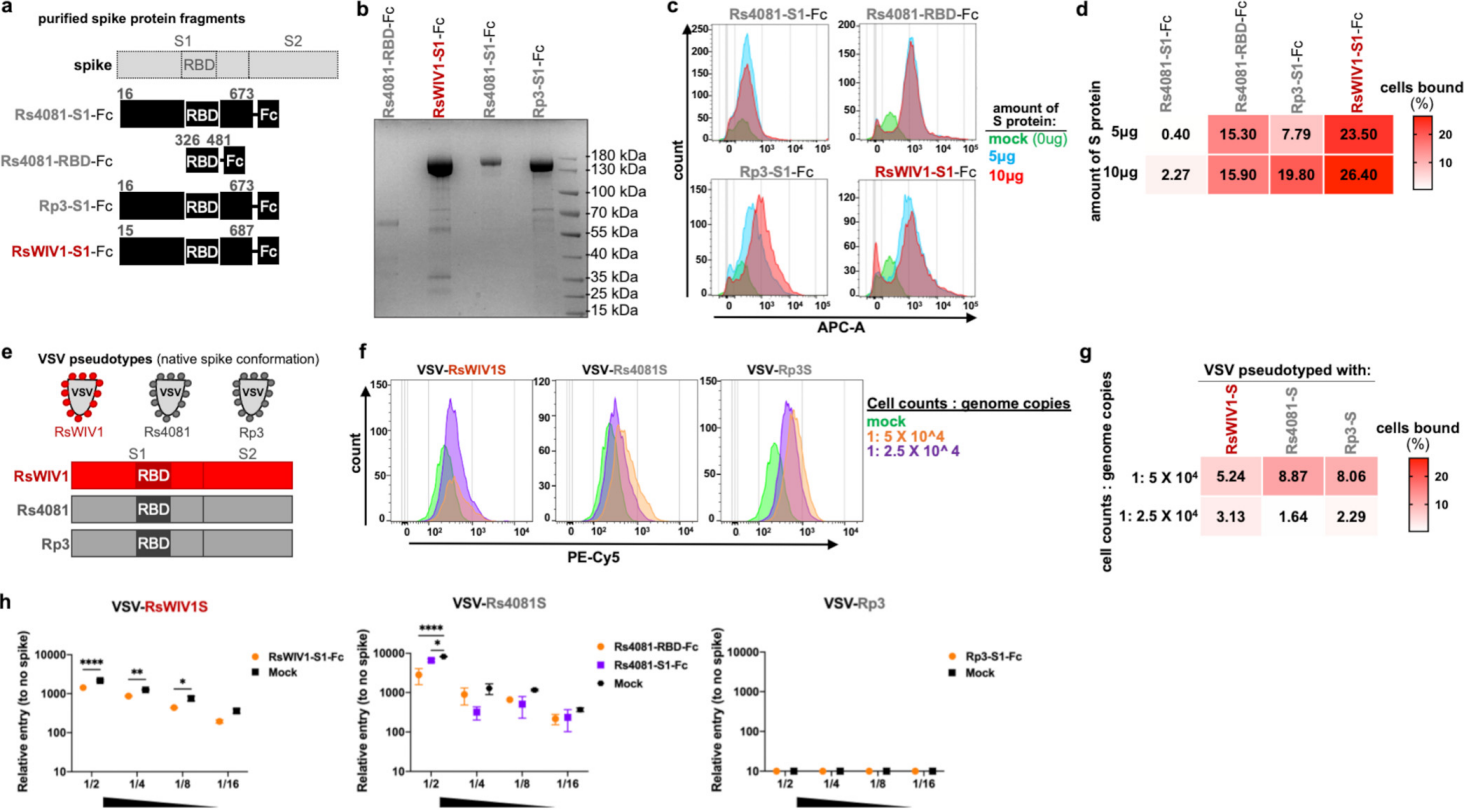
Purified clade 2 virus RBD binds to human cells. (a) Schematic overview of spike fragments used in binding assays. (b) Expression of spike fragments in HEK 293T/17 cells. (c) Purified spike fragments were incubated with Huh-7 cells at the indicated concentration, and binding was measured by FACS. (d) Heatmap representation of the percentage of cells bound by spike protein from the FACS binding data. (e) Schematic overview of pseudotyped particles bearing different spikes used in binding assays. (f) Purified pseudotyped particles were incubated with Huh-7 cells at the indicated concentration, and binding was measured by FACS. (g) Heatmap representation of the percentage of cells bound by pseudotyped particles from the FACS binding data. (h) Huh-7cells were incubated with 50 μg/mL RBD/S1-Fc proteins or DMEM (mock) at 37°C for 60 min before inoculating with 2-fold serial diluted pseudotyped stocks. Error bars represent the corresponding mean ± SEM. ****, *P* < 0.0001; **, *P* = 0.0017; *, *P* < 0.05.

In our earlier viral pseudotyped transductions and replication-competent virus infection experiments, exogenous trypsin was required in the media during the whole process, especially for the recombinant viruses that replicate in the targeted cells beyond just one cycle of cell entry. We wondered if trypsin treatment was a requirement for clade 2 virus RBD to bind cells. Having demonstrated the Huh-7 cell line is permissive for clade 2 virus infection and replication, we next tested if the S1 half of spike or the RBD of clade 2 virus could bind to the surface of Huh-7 cells. We expressed and purified spike S1 or RBD protein fragments from clade 2 viruses Rs4081 and Rp3, as well as the clade 1 virus, RsWIV1 (Fig. 3a and b), and incubated different protein concentrations with Huh-7 cells. We found that the Rs4081 RBD and Rp3 S1 protein showed relatively high binding affinity to the cells in the absence of exogenous trypsin, albeit slightly lower than clade 1 virus RsWIV1 S1 protein (Fig. 3c, d). When we treated target cells with different concentrations of trypsin and then incubated them with different viral S1 or RBD proteins, the binding affinity was significantly reduced between viral proteins and cells. Even at lower concentrations observed for other coronaviruses, 2 μg/mL of trypsin was more than sufficient to notably disrupt binding (Fig. S2).

10.1128/mbio.02566-22.2 FIG S2 Trypsin does not facilitate cell binding for clade 2 RBDs. (a and b) Purified spike fragments (2.5 μg) were incubated with cells with or without trypsin, and binding was measured by FACS. (a) Huh-7, (b) 293T, (c and d) Heatmap representation of FACS binding data, (c) Huh-7, and (d) 293T. Download FIG S2, JPG file, 0.8 MB. Copyright © 2022 Guo et al. 2022 Guo et al. https://creativecommons.org/licenses/by/4.0/ This content is distributed under the terms of the Creative Commons Attribution 4.0 International license.

Because purified S1 or RBD protein fragments are not truly representative of the multiple spike proteins found on the surface of virus particles, we repeated the binding assay by VSV-based pseudotyped particles bearing clade 1 or clade 2 virus S proteins (Fig. 3e), in Huh-7 cells. Consistent with the results from purified spike S1 or RBD protein fragments, we observed that pseudotyped particles belonging to both clades 1 and 2 exhibited concentration-dependent binding to Huh-7 cells in the absence of exogenous trypsin (Fig. 3f and g). To evaluate if purified S1 or RBD protein fragments are binding specifically to target cell receptors or nonspecifically binding cells, we incubated the Huh-7 cells with purified S1 or RBD protein fragments from clade 1 (RsWIV1) or clade 2 (Rs4081 and Rp3) at 37°C for 1 h, and subsequently transduced with serial diluted viral pseudotyped stocks in the presence of exogenous trypsin. As expected, we found that the entry of clade 1 virus RsWIV1 was significantly affected by RsWIV1 S1 protein preincubation (Fig. 3h). The entry of clade 2 virus Rs4081 also showed a decrease in the presence of Rs4081 RBD and S1 protein, albeit not as significant as clade 1 virus RsWIV1 (Fig. 3h). The other clade 2 virus, Rp3, which we have shown does not enter human cells, did not show any entry in any dilution (Fig. 3h).

Taken together, these results demonstrate that the putative clade 2 virus RBD can directly bind to some unknown molecule(s) present on the surface of Huh-7 cells. Importantly, although trypsin increases clade 2 virus entry and replication, exogenous trypsin was not required for clade 2 virus RBD cell-surface binding.

### Known coronavirus receptors are not the receptor for clade 2 sarbecoviruses.

**FIG 4:**
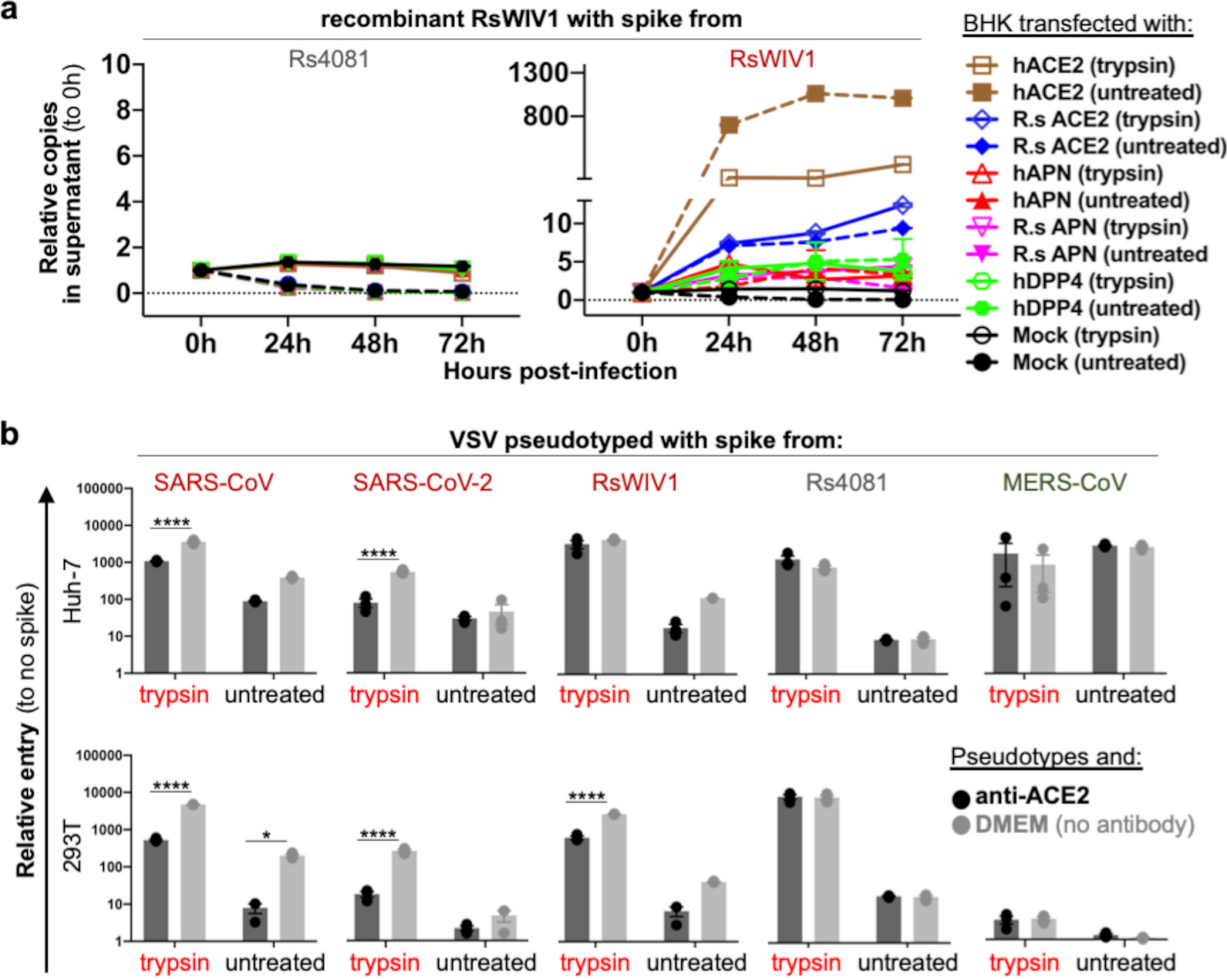
Known coronavirus receptors do not support Sarbecovirus RBD clade 2 infection. (a) Cells were transfected with the human or bat orthologues of known coronavirus receptors and infected with virus. (b) Cells were incubated with an antibody directed toward ACE2 and subsequently infected with viral pseudotyped. Error bars represent the corresponding mean ± SEM. *, *P* = 0.0332; ****, *P* < 0.0001.

To further explore the receptor usage of clade 2 sarbecoviruses, we transfected cells with human or Rhinolophus bat orthologues of known coronavirus receptors and then infected them with our chimeric viruses (Fig. 4a). We found that only the receptor for clade 1 virus, ACE2, mediated the entry of RsWIV1 in the absence and presence of trypsin, but not receptors for MERS-CoV (DPP4) or HCoV-229E (APN). In contrast, none of these receptors mediated entry of clade 2 virus Rs4081, even in the presence of trypsin (Fig. 4a). To further test if ACE2 was involved specifically in the human cell lines we used in our previous experiments, we incubated HEK 293T and Huh-7 cells with anti-human ACE2 antibody and subsequently infected them with viral pseudotyped (Fig. 4b). Attempts to block ACE2 only significantly reduced the entry of clade 1 spikes, SARS-CoV, SARS-CoV-2, and RsWIV1, but not Rs4081 or MERS-CoV (Fig. 4b).

We also tested several alternative receptors that have been described for SARS-CoV-2. Overexpression of neuropilin-1 (NRP1), AXL, KREMEN1, or ASGR1 in BHK cells failed to recover entry of any clade 1 or 2 sarbecovirus, regardless of trypsin, further confirming that clade 2 sarbecoviruses employ an entry route in human cells that is distinct from other coronaviruses (Fig. S3).

10.1128/mbio.02566-22.3 FIG S3 Alternative SARS-CoV-2 receptors do not mediate RBD clade 2 cell entry. BHK cells were transfected with alternative SARS-CoV-2 receptors (a) AXL or NRP1, (b) ACE2, ASRG1, and KREMEN1, and infected with viral pseudotyped. Download FIG S3, JPG file, 0.5 MB. Copyright © 2022 Guo et al. 2022 Guo et al. https://creativecommons.org/licenses/by/4.0/ This content is distributed under the terms of the Creative Commons Attribution 4.0 International license.

## DISCUSSION

Trypsin and other extracellular proteases play an essential role in metabolism regulation and digestion and are found in abundance within the intestinal lumen (35). Early attempts to isolate enteric coronaviruses, such as porcine endemic diarrhea coronavirus (PEDV) and enteropathic bovine coronavirus, failed, even in cell cultures derived from the host species, and were only successfully propagated when trypsin was included in the culture medium (36 to 38). Similarly, here trypsin also fully mediated clade 2 sarbecovirus entry in both human and bat cells (Fig. 1). Of note, only some of the clade 2 viruses efficiently replicated in culture, with Rs4081 replicating similar to wild-type clade 1 virus RsWIV1 (Fig. 1c). Recently, we further mapped trypsin-dependent entry for clade 2 viruses to a putative receptor-binding motif encompassing Rs4081 amino acids 405 to 481 and showed that exchanging this region between clade 2 spikes could toggle trypsin-dependent entry (20). Altogether, our findings with replication-competent virus further demonstrate that trypsin-dependent clade 2 sarbecovirus entry in human cells is both virus and cell specific.

Our group identified bat sarbecoviruses, including all of the clade 1 and 2 viruses used in this study, in Rhinolophus fecal samples, strongly suggesting that sarbecoviruses naturally replicate in the bat gastrointestinal system (6, 10). In support of this putative tissue tropism, Rhinolophus sinicus intestinal cells were the only bat cell line that supported efficient clade 2 virus replication (Fig. 1g). Curiously, the clade 2 sarbecoviruses exhibited a higher resistance to trypsin activation than other coronaviruses, including clade 1 sarbecoviruses (Fig. 2a and b; Fig. S1a and b), which may indirectly reflect the tissue environment of these viruses, or dependence on host species–specific protease (20). More studies are needed to determine the exact protease composition within the bat intestinal lumen.

**FIG 5:**
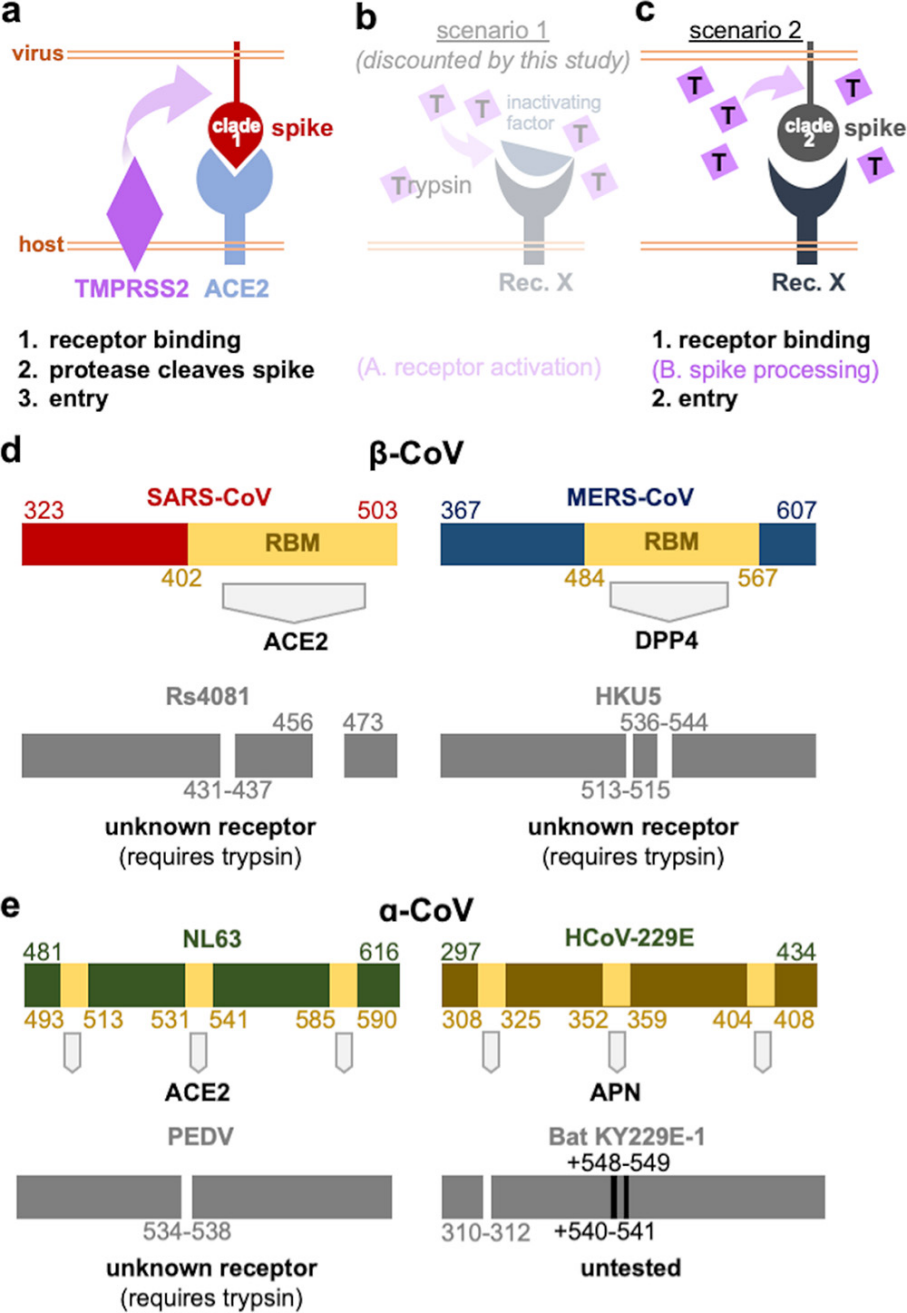
Essential role for exogenous protease in CoV entry. (a) Surface-bound host-cell protease mediates clade 1 RBD entry following ACE2 binding. (b) Data from this study exclude the possibility that trypsin may act on the receptor and support the possibility (c) that trypsin cleaves viral spike during entry. (d) Trypsin-dependent, bat-derived betacoronaviruses contain deletions in their RBDs compared to closely related coronaviruses with known receptors. (e) Alphacoronaviruses with known receptors also differ from a subset of closely related but trypsin-dependent, animal viruses with deletions or insertions in their RBDs.

After binding the host receptor, many coronaviruses, including SARS-CoV-2 and MERS-CoV, rely on the host protease TMPRSS2 found at the cell surface or cathepsin B/L in the endosome, to cleave viral spike, releasing the fusion peptide and mediating cell entry (Fig. 5a). Although the mechanism of trypsin-enhanced coronavirus entry is still not completely known, trypsin is likely also involved in processing viral spike during entry (Fig. 5c). We can rule out the possibility that trypsin acts directly on the host cell prior to infection (Fig. 5b), because purified clade 2 virus spike RBD and viral pseudotyped with clade 2 spike in a native conformation could bind human cells without trypsin, while pretreating target cells with trypsin had no measurable effect on virus RBD cell-surface binding (Fig. 3; Fig. S2). Purified spike protein could also reduce RBD clade 2 entry, further suggesting trypsin is not directly involved in receptor engagement (Fig. 3h). Trypsin has been shown to compensate for TMPRSS2 for some coronaviruses, but not all. For example, while PEDV replicates in cells expressing TMPRSS2, betacoronaviruses such as the merbecovirus, HKU5, and PDF2180 have only replicated in the presence of trypsin, even in Caco2 cells, which endogenously express TMPRSS2 (27, 29). Analogously, clade 2 sarbecoviruses did not replicate in Caco2 cells without trypsin, or in cells overexpressing TMPRSS2 (Fig. 2c). Attempts to block TMPRSS2 with camostat only had a measurable effect on clade 1 virus RsWIV1 in Caco2 cells, but not clade 2 viruses (Fig. 2g and i). Although TMPRSS2 did not appear to be involved in clade 2 entry, drugs that blocked endosomal proteases, cathepsin B/L, did reduce clade 2 virus entry in Huh-7 cells, similar to MERS-CoV (Fig. 2h and i). Our data show that TMPRSS2 does not affect clade 2 entry, and although endosomal proteases may be involved in clade 2 entry, trypsin is still an essential requirement for these viruses. Moreover, the Rs4081 RBD still required trypsin for entry even in the SARS-CoV-2 spike, which contains a furin cleavage site purported to improve viral entry (Fig. 2d to f). These data show that a furin site does not compensate for other proteases during entry. Treating concentrated clade 2 spike pseudotyped particles with different amounts of trypsin resulted in a clear pattern of spike digestion corresponding to cleavage at the S1/S2 and S2’ junctions, as has been shown for other coronavirus spike proteins (Fig. S1f to h) (39 to 45). Our results showing that prolonged incubation with high levels of trypsin completely ablated the spike protein within 15 min suggest that trypsin is likely acting on spike rapidly following receptor engagement (Fig. S1f). Thus, while trypsin is often used in coronavirus cell culture to overcome missing or incompatible host proteases needed for viral spike processing and entry, our findings suggest exogenous protease may, itself, be an essential cofactor for ACE2-independent sarbecoviruses. Sapoviruses, predominantly gastrointestinal pathogens, were recently shown to require bile acids for replication *in vitro*, further adding to this notion that viruses adapt to their extracellular environment (46).

Our data add to a growing trend observed for coronaviruses with a trypsin requirement for entry. RBDs from clade 2 sarbecoviruses are shorter than clade 1 viruses like SARS-CoV and require trypsin for entry but do not use the clade 1 receptor ACE2 from any species (Fig. 4 and 5d). RBDs from merbecoviruses HKU5 and PDF2180 are shorter than MERS-CoV, require trypsin for entry, and do not use the MERS-CoV receptor (Fig. 5d). RBDs from alphacoronaviruses are tripartite, and some of them even interact with the same receptors as betacoronaviruses. In a similar fashion, RBDs from enteric alphacoronaviruses such as PEDV are shorter than other alphacoronaviruses, require trypsin, and do not use the known receptors for cell entry (Fig. 5e). Our group identified sarbecoviruses cocirculating in high abundance in bat populations and occasionally found more than one sarbecovirus per sample. Given that recombination is a driver of sarbecovirus diversity and only occurs when more than one virus infects the same cell, it is likely that clade 1 and 2 viruses infect similar cell types and tissues in bats. In an environment rich with genetically similar viruses all competing for the same host resources, there is likely strong selective pressure for shifts in cell tropism. Thus, while ACE2 usage may be considered an evolvable trait among clade 1 viruses that already possess basal capacity to use the receptor, ACE2 usage in general appears to also be a completely dispensable trait for the subgenus (37, 38, 47 to 49). How adaptable receptor-X usage is among the ACE2-independent sarbecoviruses remains to be determined, but our experiments exchanging the RBM between these viruses show this trait is confined to a viral sequence and should therefore be adjustable (20).

Our findings that clade 2 sarbecoviruses may specifically require trypsin for replication further suggest they are enteric in their hosts. Similarly, SARS-CoV, SARS-CoV-2, and MERS-CoV have all been shown to replicate in the gastrointestinal tract and shed in the feces of infected patients, and fecal–oral transmission has been shown as an alternative route of exposure for these predominantly respiratory pathogens (50). Therefore, there may still be a risk of transmission of viruses that are more strictly enteric. Taken together, we have demonstrated yet another group of betacoronaviruses with zoonotic potential, which will be instrumental in developing broader coronavirus vaccines. More importantly, this work provides evidence for what may be a widely employed mechanism for betacoronavirus entry and will serve as a road map for future studies on clade 2 RBD sarbecoviruses.

## MATERIALS AND METHODS

### Cells.

Bat-derived cell lines RSI9410, RSL4323, and RfKT were generated and stored at the Wuhan Institute of Virology as previously described (51). RSI9410 and RSL4323 were derived from intestine and lung tissues from Rhinolophus sinicus, respectively. RfKT cells were derived from Rhinolophus ferrumequinum kidney tissue and further immortalized through stable overexpression of the SV40 T-antigen. HEK 293T, HEK 293T/17, and BHK-21 (obtained from the American Type Culture Collection [ATCC]), Caco2 (generously gifted by Qin-Xue Hu), and Huh-7 cells were maintained in Dulbecco's modified Eagle medium (DMEM) supplemented with 10% fetal bovine serum (FBS). Bat cells were maintained in Dulbecco's Modified Eagle Medium/Nutrient Mixture F-12 supplemented with 15% fetal bovine serum (FBS). Cultures were maintained at 37°C with 5% CO_2_. All cell lines used in this study were species verified by cytochrome sequencing and tested negative for mycoplasma contamination by PCR as described previously (11, 19).

### Plasmids.

Expression plasmids for human ACE2, human DPP4, human APN, Rhinolophus sinicus ACE2, and the infectious cDNA clones of SARSr-CoV RsWIV1 with spike genes from bat SARSr-CoV Rs4081, As6526, Rs4235, Rf4092, Rs4247, or Rp3 S gene have been described elsewhere (10, 11, 15, 52). Human ASGR1 and KERMEN1 genes were amplified from the Hep G2 cell line using the reported primers (53). The Rhinolophus sinicus APN was amplified from the bat intestine as described previously (52), and the primers used for the amplification were as follows: first-round F-APN-out: 5′-CCTCCGGGATATAAGCCTG-3′; first-round R-APN-out: 5′-ACAGGCAGAGGGGAGAGG-3′; second-round F-APN-in: 5′-ATTT*GCGGCCGC*GCCACCATGGCCAAGCCCCTCG-3′; second-round R-APN-in: 5′-CTA*GCTAGC*TTGGCTGTGGTCTGTGAAC-3′. The human ASGR1, human KERMEN1, and Rhinolophus sinicus APN genes were cloned into a pCAGGS expression vector with an N-terminal signal peptide and a C-terminal Stag followed by a stop codon. CMV-driven expression plasmids for human AXL (GenBank accession number: AAH32229) with a C-terminal FLAG tag were acquired from Addgene (originally sourced from Rosa Melillo lab: https://www.addgene.org/105933/); for human neuropilin-1 (GenBank accession number: NM_001024628.2) with C-terminal FLAG were obtained from SinoBiological (cat.: HG10011-CF); and for human TMPRSS2 (GenBank) were obtained from Addgene (plasmid number 53887). Bat SARSr-CoV spikes were described previously (20). All the plasmids used in this study were verified by Sanger sequencing.

### Recovery of live virus.

Recombinant viruses were rescued by two strategies (see Fig. 1b), as described previously, with minor adjustment (10, 29, 54, 55). In brief, BHK-21 cells were seeded in a 6-well-plate and transfected with 6 μg infectious clones at a cell confluence of 70%, using Lipofectamine 3000 (Invitrogen) according to the manufacturer’s instructions. At 6 h posttransfection, culture medium was replaced with serum-free DMEM. At 24 h posttransfection, cells were either cultured for another 48 h or trypsinized, plated over a 50% confluent monolayer of Huh-7 cells, and cocultured for another 48 h. The cell-free supernatant was harvested and mixed with the same volume of cold trypsin or DMEM (untreated) to a final concentration of 100 μg/mL trypsin, before inoculating to the target cells in a 24-well-plate. The inoculated plates were centrifuged at 1,200 *g* for 1 h at 4°C and then incubated in a 37°C incubator for 72 h. Culture supernatants were collected at 0, 24, 48. and 72 h postinfection, with 50 μL supernatant at each time point, and stored at −80°C for future use. The viral RNA of recombinant viruses used in this study was verified by next-generation sequencing (NGS). The trypsin used for viral pseudotyped transduction and replication-competent virus infection experiments is a mixture of trypsin and chymotrypsin without EDTA and not TPCK-treated (Thermo Fisher Scientific, 15050057).

### Protein expression and purification.

The constructs used for protein expression were prepared as previously described with minor adjustments (15, 52). In brief, the codon-optimized genes encoding the RsWIV1-S1 (spike residues: aa15-687, accession number: AGZ48828), Rs4081-S1 (spike residues: aa16-673, accession number: ATO98120), Rs4081-RBD (spike residues: aa326-481, accession number: ATO98120), and Rp3-S1 (spike residues: aa16-673, accession number: AAZ67052) were synthesized by Sangon Biotech (Shanghai, China) and inserted into a modified pCAGGS expression vector with an N-terminal signal peptide and a C-terminal human IgG Fc tag followed by a stop codon. The proteins used for the binding assay were expressed in HEK 293T/17 cells as described previously (15, 52), purified by protein A/G agarose (Thermo Scientific), and eluted by IgG Elution Buffer (Thermo Scientific) according to the manufacturer’s instructions. Purified proteins were buffered in PBS, quantified by a Qubit 2 Fluorometer (Thermo Scientific), aliquoted, and stored at −80°C for further use.

### Pseudotyped virus production and entry assay.

The coronavirus spike pseudotyped particles with VSVΔG-luc/GFP backbone were generated as previously described (19). For entry assays, target cells were seeded in a 96-well plate and washed with PBS once before inoculating with different pseudotyped stocks. For trypsin conditions, the same volume of cold trypsin or DMEM (untreated) was added to the pseudotyped stocks to a final concentration of 100 μg/mL. The trypsin or untreated conditions were subsequently inoculated on the target cells. Inoculated plates were centrifuged at 4°C at 1,200 *g* for 1 h and then incubated at 37°C for 18 to 20 h. Entry efficiency was quantified by measuring the luciferase activities using Bright-Glo luciferase reagent (Promega), following manufacturer’s instructions. For receptor usage assays, BHK-21 cells were transfected with plasmids expressing ACE2, DPP4, APN, AXL, ASGR1, KERMEN1, and NRP1 18 to 24 h before transducing with different pseudotypes (19). To test the protease inhibitors, target cells were pretreated with 25 μM E-64d (Sigma-Aldrich, E8640), 100 μM camostat mesylate (MedChemExpress, HY-13512), and 25 μM E-64d + 100 μM camostat mesylate or DMEM + 10% FBS (mock) at 37°C for 2 h before incubating with pseudotyped stocks. For the anti-ACE2 blocking assay, target cells were preincubated with 20 μg/mL anti-ACE2 antibody (R&D Systems, goat, AF933) or DMEM (mock) at 37°C for 30 min before inoculating with different pseudotyped stocks as previously described (31). For the RBD/S1 protein blocking assay, target cells were preincubated with 50 μg/mL RBD/S1-Fc proteins or DMEM (mock) at 37°C for 60 min before inoculating with 2-fold serial diluted pseudotyped stocks as described above. All entry assays with pseudotypes were independently repeated more than twice with at least three technical replicates in each experiment. Relative entry was calculated as the fold entry over the negative control, by normalizing the relative luciferase activities for spike pseudotyped to the no-spike control.

### Real-time PCR.

Viral RNA was extracted as previously described and converted to cDNA using HiScript II One Step qRT-PCR SYBR green kit (Q221-01, Vazyme) (52). Viral replication in HEK 293T, Caco2, Huh-7, RSI9410, RSL4323, RfKT, and transfected BHK-21 cells was quantified by RT-PCR using primers targeting the RdRp gene of bat SARSr-CoV RsWIV1 (52, 56). The titer of RsWIV1 was determined as previously reported (52), and the RNA from RsWIV1 stocks with known titer was used as a standard control to correlate *C_T_* value and virus titer of clade 2 viruses. Quantification of pseudotyped particles by real-time PCR was performed as previously described (15). All samples were analyzed in duplicate on two independent runs. One representative data set is shown.

### Flow cytometry.

To analyze the replication of recombinant virus in target cells, infected cells were fixed by 4% paraformaldehyde (PFA) at room temperature (about 25°C) for 30 min after 48 h postinfection. Cells infected with virus stocks without trypsin were trypsinized before fixation. Expression of sarbecovirus N protein was detected by flow cytometry using rabbit serum against the SARSr-CoV Rp3 N protein followed with fluorescein isothiocyanate (FITC) labeled goat anti-rabbit antibody (Abcam, ab6717), as previously reported (6, 52).

For binding assays, Huh-7 cells were scraped by a cell scraper, washed twice with PBS, and diluted to 3 × 10^5^ cells/condition before incubating with different S1 or RBD proteins at 37°C for 30 min, with or without trypsin. Binding between proteins and cells was detected by a Dylight 650 labeled goat anti-human IgG Fc antibody (Abcam, ab98622). For pseudotyped particles binding assay, the pseudotypes were generated as described above and purified through ultracentrifugation as previously described (57). The purified pseudotyped particles were incubated with Huh-7 cells at 4°C for 1 h before fixation by 4% PFA and permeabilization by PBS plus 0.25% Triton X-100. Binding between purified pseudotyped particles and cells was detected by a mouse anti-FLAG antibody targeting the C-terminal spike, following by a Cy3-labeled goat anti-mouse IgG antibody (Abcam, ab97035).

### Statistical analysis and graphing.

Statistical significance for entry assays was determined by 2-way analysis of variance, with Sidak test correction for multiple comparisons, using GraphPad Prism 9.

### Biosafety and biosecurity.

Laboratory work with VSV pseudotypes in mammalian cell lines was performed according to standard operating procedures (SOPs) under biosafety level 2 (BSL2) conditions that were approved by institutional biosafety committees (IBC) at Washington State University and the Wuhan Institute of Virology (WIV). Works with the attenuated infectious viral clone RsWIV1 (56) and clade 2 virus were approved by the WIV IBC for this SOP. The institutional facilities were designed to conform to the safety requirements recommended by Biosafety in Microbiological and Biomedical Laboratories (BMBL), the China National Accreditation Service for Conformity Assessment.
