# Supplementary figures and images for: ACE2-Independent Bat Sarbecovirus Entry and Replication in Human and Bat Cells
Source: mBio. 2022 Nov 21;13(6):e02566-22. doi: 10.1128/mbio.02566-22 (PMC9765407; doi:10.1128/mbio.02566-22)

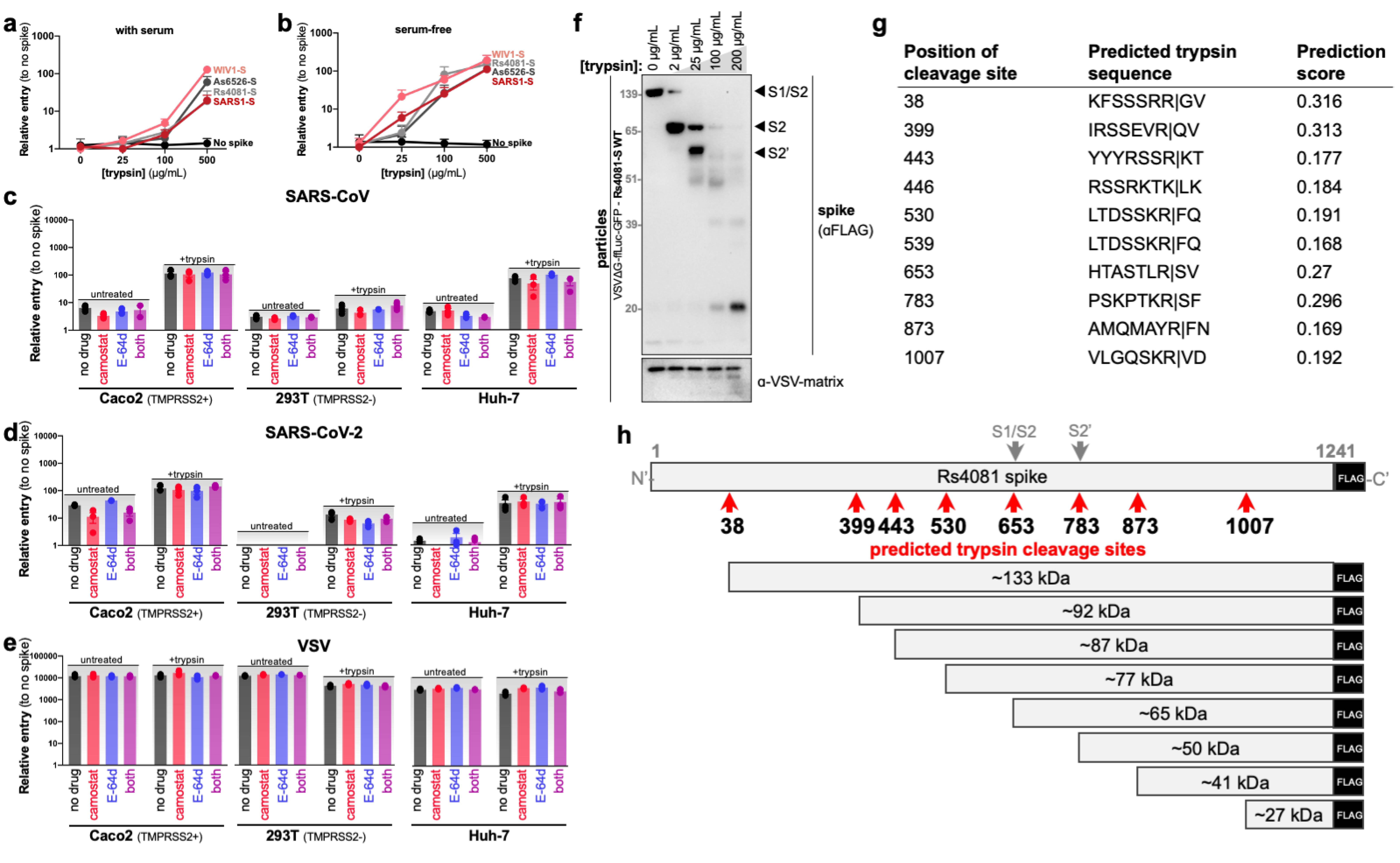

Supplement: FIG S1 [file mbio.02566-22-s0001.jpg]

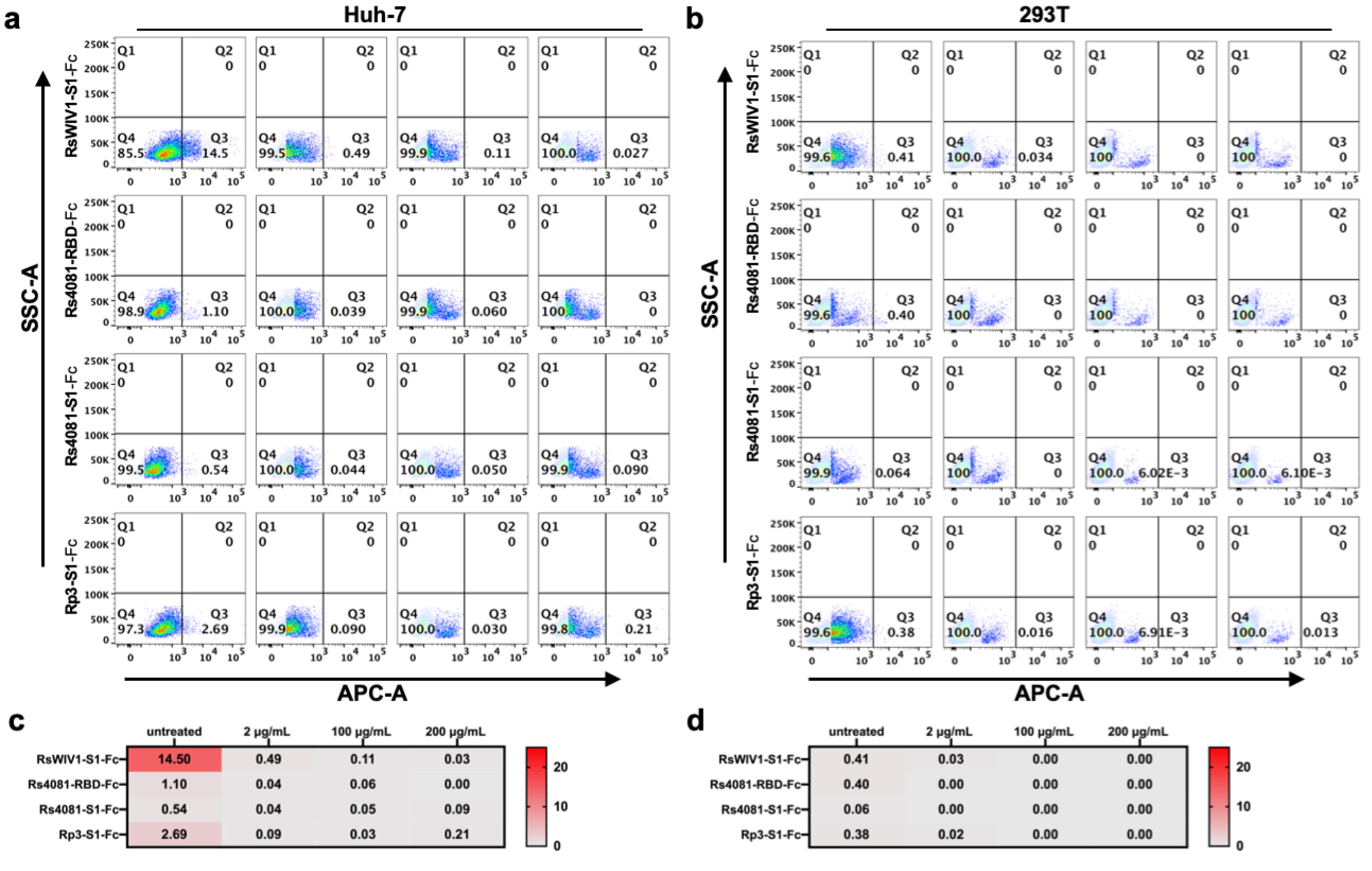

Supplement: FIG S2 [file mbio.02566-22-s0002.jpg]

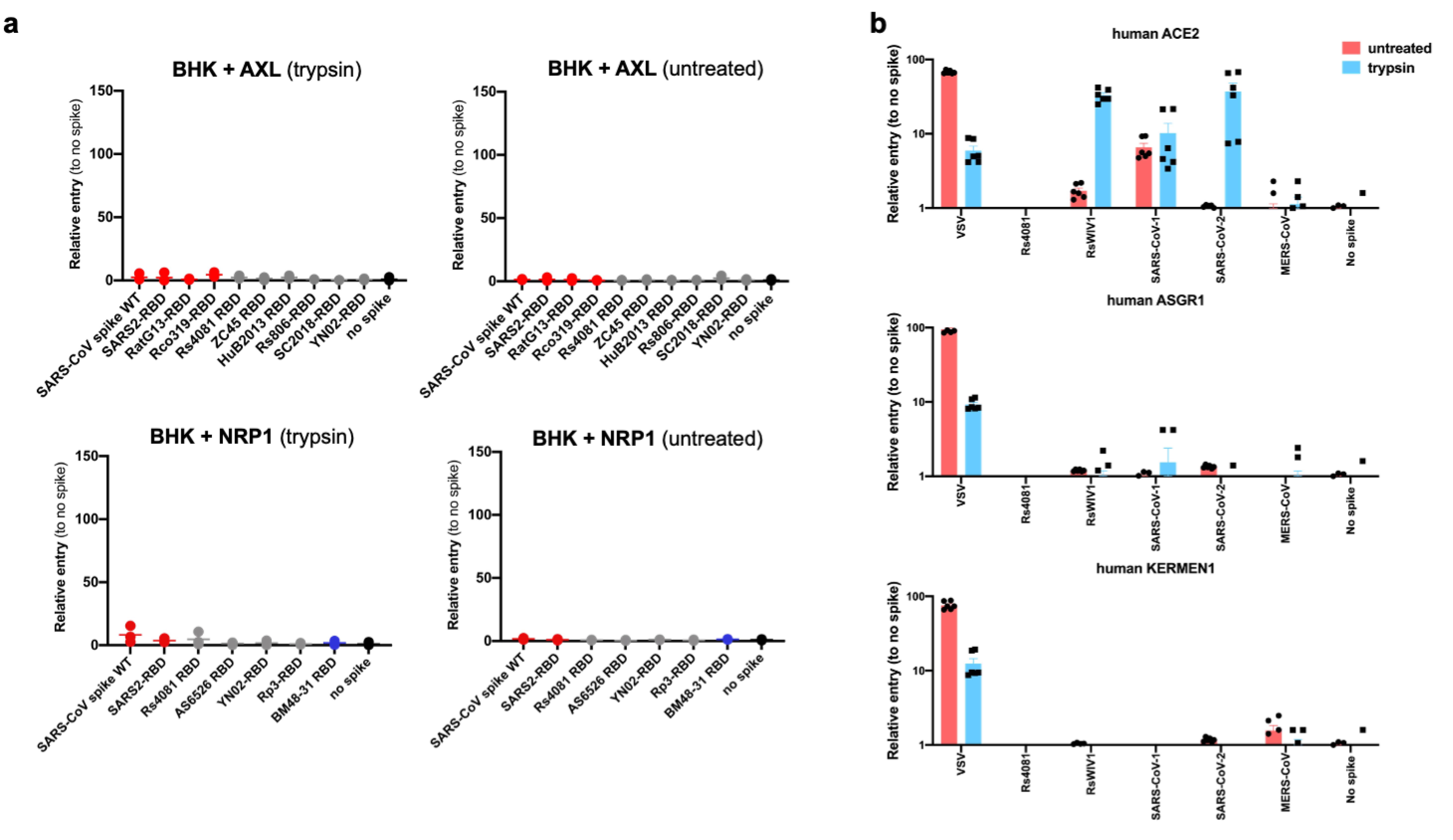

Supplement: FIG S3 [file mbio.02566-22-s0003.jpg]
